# Household and social characteristics associated with COVID-19 vaccine intent among Latino families in the San Francisco Bay Area

**DOI:** 10.1186/s12879-022-07467-3

**Published:** 2022-06-07

**Authors:** Janet M. Wojcicki, Milagro Escobar, Andrea DeCastro Mendez, Suzanna M. Martinez

**Affiliations:** 1 Division of Pediatric Gastroenterology, Hepatology and Nutrition, Department of Pediatrics, UCSF, 550 16th Street, San Francisco, CA 94134-0136 USA; 2 Department of Epidemiology and Biostatistics, UCSF, San Francisco, USA

**Keywords:** COVID-19, Latino children, Vaccine uptake, Nonintent

## Abstract

**Background:**

Latinos have had higher case counts, hospitalization rates and deaths during the COVID-19 pandemic nationally and in the state of California. Meanwhile, Latino vaccination rates remain lower than those of non-Hispanic Whites. COVID-19 vaccine nonintent, defined as intent to not vaccinate against COVID-19, among Latino individuals continues to be an issue in the state of California.

**Methods:**

Families from three Latino longitudinal mother–child cohorts previously recruited in the San Francisco Bay Area were surveyed telephonically from February to June 2021 to assess attitudes towards vaccination against COVID-19 and prior vaccination, in general, for themselves and their children. Risk for vaccine nonintent was assessed using the Mann–Whitney rank sum non-parametric test for continuous predictors and chi-squared tests for categorical ones.

**Results:**

Three hundred and nineteen families were surveyed from the Telomere at Birth (TAB), Hispanic Eating and Nutrition (HEN) and Latino Eating and Diabetes Cohort (LEAD). Approximately 36% from TAB and 28% from HEN/LEAD indicated COVID-19 vaccine nonintent for themselves and/or their children. Risk factors for vaccine nonintent included lower maternal age (p = 0.01), concern about vaccine side effects (p < 0.01) and prior history of a household members being infected with SARS-CoV-2 (p < 0.01) and indexes of household crowding including number of people sharing a bathroom (p = 0.048). Vaccine intent was also associated with receiving vaccine input from friends (p = 0.03), family (p < 0.01) and/or coworkers (p = 0.02) compared with those who were not planning on getting vaccinated against COVID-19.

**Conclusions:**

Latino families living in crowded living situations who may not have received any COVID-19 advice from family, coworkers or friends are at particular risk for nonintent for vaccinatation against COVID-19. Community-based grassroots or *promotor/a* based interventions centered on trusted individuals with close community ties and counseling concerning vaccination against COVID-19 could help boost vaccination rates in this population group.

**Supplementary Information:**

The online version contains supplementary material available at 10.1186/s12879-022-07467-3.

## Background

### COVID-19 morbidity and mortality

The COVID-19 pandemic has resulted in over 507 million cases worldwide and 6.2 million confirmed deaths since emerging on the global scale in 2020 [1]. Worldwide, there have been more than 11 billion doses of the vaccine against COVID-19 administered [1].

As of April 2022, there have been over 988,000 deaths from COVID-19 in the United States with high rates among racial and ethnic minority groups [2]. Over the course of the pandemic, Latinos have been two times more likely to become infected with SARS-Co-V2, the coronavirus that causes COVID-19 disease, 2.3 times more likely to have hospitalization, and 1.8 times more likely to die after adjusting for population age structure, in comparison to White, Non-Hispanic groups [3]. Latinos account for 18.5% of the US population but 24.9% of COVID-19 cases [2]. The rates of COVID-19 and mortality are likely higher among undocumented Latinos but immigration status is not routinely collected by states or counties [4].

### Vaccination against COVID-19 among Latinos

Meanwhile, rates of vaccination against COVID-19 among Latinos are lower than the overall proportional representation of Latinos in the United States. Only 15.1% of eligible Latinos have recieved booster doses (with the mRNA Pfizer/BioNTech or Moderna vaccine after receiving two doses of either or one dose of the Johnson and Johnshon/Jassen) while Latinos represent 19.6% of the eligible population [5]. In California, approximately 40% of Latinos that are eligible have not completed the primary vaccination series against COVID-19 and less than 50% who are eligible have received a booster [6]. In particular, among eligible 5–11 year olds only 22.8% of Latino children have been vaccinated compared with 28.0% of non-Hispanic White and 64.2% of Asian children in this age category [6]. Meanwhile, the burden of disease has also been higher in Latinos compared to other population groups in the state of California. Overall, Latinos have been 47.5% of cases in California and 43.7% of deaths but they represent only 38.7% of the population in California [7].

### Reasons for nonintent to vaccinate against COVID-19 among Latinos

In the state of California, vaccination is provided to all individuals over the age of 5 without any need for payment or verification of immigration status. Vaccination without need for payment was instituted in California as of April 15, 2021. Vaccination intent is defined as being absolutely certain or likely to be vaccinated [8]. Racial and ethnic minorities have a greater rate of nonintent for vaccination against COVID-19 (plan not to vaccinate against COVID-19). A recent review combined data from 13 studies on vaccine intent for COVID-19 finding that vaccine hesitancy was much higher for Latinos (41.6%) versus 26.3% for all other adult Americans [9]. A study focused on vaccine hesitancy among Latino adults found that adults younger than 50 were more likely to be distrustful of public health authorities than older Latino adults [10].

Factors that may impact vaccine intent in Latino communities include lack of legal status to minimal prior history with US healthcare services, wariness of authorities, and cultural and linguistic barriers [11]. In particular, there has not been sufficient inclusion of the Latino community or collaboration with Latino community resources during public health planning for vaccination roll-out against COVID-19 [11].

As an increasing percentage of the population gains some immunity to SARS-Co-V2 through vaccination (77.5% having received one vaccination dose as of April 2022, [12]), COVID-19 infection and hospitalization rates are primarily among the unvaccinated [13]. As such, there is increased urgency to better understand nonintent for vaccination against COVID-19 among Latino communities. The present study examined vaccine nonintent and the intention for vaccine uptake against COVID-19 among three pre-existing longitudinal cohorts of Latinos residing in the Greater San Francisco Bay Area.

## Methods

### Latino cohorts and recruitment

In Spring 2021, in response to the availability of vaccines against COVID-19 in the United States (specifically two type of mRNA vaccines, Moderna and Pfizer/BioNTech and one viral vector vaccine, the Janssen or J&J vaccine), we surveyed three existing Latino mother–child, family-based longitudinal cohorts in the San Francisco Bay Area, the Hispanic Eating and Nutrition (HEN), Latino Eating and Diabetes (LEAD) study and the Telomeres at Birth (TAB) cohort. In short, these Latino cohorts were recruited initially as mother–child birth cohorts at UCSF Benioff and Zuckerberg San Francisco General (ZSFG) hospitals to evaluate early life risk factors for childhood obesity. Details about these cohorts have been previously described including methods of recruitment and demographics [14, 15]. In short, all mothers resided in San Francisco at the time of recruitment, and children were born at UCSF or ZSFG hospitals. The HEN and LEAD cohorts consist primarily of foreign-born mothers (from Mexico and Central American countries) and were recruited primarily from ZSFG whereas TAB includes a more heterogenous mix of Latino families recruited primarily from UCSF Benioff. HEN and LEAD participants had lower levels of education and tended to participate in the Special Supplemental Program in Women and Infant Nutrition (WIC) more than participants from TAB [14, 15]. We re-contacted these cohorts telephonically in 2020 during an early period in the COVID-19 pandemic (May to September 2020) to assess food insecurity (as measured by 18 questions from the US Food Security Food Module (US HFSSM) [16]), housing conditions (including how many individuals living in a household, number of individuals sharing a bedroom and sharing a bathroom) and risk factors for COVID-19 infection (e.g. as measured by working as an essential worker during lockdown) [17, 18].

### Vaccination against Covid-19 survey

Subsequently, we re-contacted families to assess attitudes towards vaccination against COVID-19 in February-June 2021 once vaccinations had received emergency US Food and Drug Administration (FDA) approval (Pfizer-BioNTech, Moderna and Johnson & Johnson Janssen) in the United States. The FDA initially approved Pfizer-BioNTech for adult vaccination against COVID-19 on December 11, 2020 [19]. Subsequently, the FDA authorized children 16 to 17 years of age to receive emergency approval on May 10, 2021 [20]. Due to those policy changes at the end of 2020 and early 2021, we surveyed participants about vaccination history for themselves and their children, intent to vaccinate against COVID-19 and intent to vaccinate their children against COVID-19 (once approval was given for their children’s age group for vaccination against COVID-19) using a short 10-min questionnaire administered telephonically (see Additional file 1: supplements). We additionally surveyed participants regarding those individuals and institutions including media sources that had possibly influenced their attitudes toward vaccination against COVID-19. We also asked participants two open-ended questions about what could change their willingness to get vaccinated against COVID-19 (1) for themselves and (2) for their children. All participants from the original cohorts who continued to reside in the United States were eligible to participate. Participants who were currently living in Mexico, Central America or another international context were not surveyed. Participants were interviewed in English or Spanish based on language of choice by trained research assistants and the principal investigator of the study.

### Data analysis

We combined the HEN and LEAD cohorts for analyses purposes due to similar inclusion criteria (Latina mothers anticipating a healthy newborn) and methods of recruitment primarily at the ZSFG hospital were similar [14, 15]. TAB was analyzed separately. Demographic and socioecononomic factors including age, household size and household density, food security and employment status as well as past vaccination history were assessed in relationship to self-disclosed nonintent for vaccination against COVID-19 for self and/or child. The following categories based on responses to the United States Household Food Security Survey Module (US HFSSM) [16] were used to indicate food security: high food security or food secure (0 affirmative responses), marginal food security (1–2 affirmative responses), low food security (3–7 affirmative responses), and very low food security (8–18 affirmative responses).

We also assessed differences in influences that impacted intent to vaccinate against COVID-19 between those who intended or had already vaccinated and those who were not planning to be vaccinated. We compared groups using chi-squared tests and Wilcoxon rank sum tests for categorical and continuous predictors respectively. Continuous predictors were tested for normality using the Shapiro Wilk test for normality and all predictors followed non-parametric distributions. All statistical analyses were conducted using Stata 15.0. For the qualitative data, we grouped answers into themes if two or more participants raised the same issues of concern regarding intent to vaccinate against COVID-19.

## Results

### Description of cohorts

We had 211 participants from the TAB cohort who completed the vaccine intent survey and 108 from the HEN/LEAD cohorts. There was a low refusal rate (< 10%) among participants who were successfully contacted telephonically. Demographics including language use and household socioeconomic status are presented by cohort in Table 1. Average household size was 5.1 ± 1.8 persons in HEN/LEAD and slightly smaller at 4.7 ± 1.9 persons in TAB. Both cohorts reported a mean of 2 to 3 children in the household (Table 1). Almost all HEN/LEAD participants reported Spanish as the primary language (93.5%) in contrast with TAB where 32.1% reported Spanish as their primary language (64.6% reported English as their primary language) (Table 1). Maternal age was slightly higher in HEN/LEAD (37.4 ± 6.8) versus 35.4 ± 5.6 in TAB. A higher percentage of TAB reported being married or living with partner (91.4%) versus 48.6% in HEN/LEAD and similarly a high percentage of TAB had a high school degree or higher (81.8%), 83.2% of HEN/LEAD had a high school diploma or less (Table 1).

**Table 1:** Population characteristics of Latinx cohorts in San Francisco Bay Area

Cohort name	Telomeres at birth (TAB) Mean ± SD (or %) (n = 211)	Hispanic, eating and nutrition (HEN) & Latino, eating and diabetes (LEAD) (n = 108)
Household specifics		
No. of people in household	4.7 ± 1.9	5.11 ± 1.82
No. of children in household	2.1 ± 0.96	2.6 ± 1.03
Languages spoken at home		
English	64.6% (135/209)	6.5% (7/107)
Spanish	32.1% (67/209)	93.5% (100/107)
Bilingual (English & Spanish)	3.4% (7/209)	N/A
Maternal age		
Age (years)	35.4 ± 5.6	37.4 ± 6.8
Maternal marital status		
Married/Living with partner	91.4% (191/209)	51.4% (55/107)
Single/Other	8.5% (17/209)	48.6% (52/107)
Maternal high school education		
Has high school diploma	81.8% (171/209)	
No high school diploma	18.2% (38/209)	
Less than or equal to high school/GED		83.2% (89/107)
Household socioeconomic status		
Number sharing bathroom (persons)	3.4 ± 1.7	4.2 ± 1.6
Number sharing bedroom (persons)	2.4 ± 1.0	2.4 ± 1.1
Household food insecurity during COVID-19 lockdowns	34.1% (63/185)	64.5% (60/93)
No adult employed in household during COVID-19 lockdowns	23.2% (43/185)	55.4% (56/83)
Ethnicity/ethnic identity and place of birth		
Mexican/Mexican–American	31.0% (64/210)	55.9% (57/102)
Central American	29.5% (62/210)	39.2% (40/102)
South American	8.1% (17/210)	4.9% (5/102)
Caribbean	1.9% (4/210)	N/A
Spain/Portugal	6.7% (14/210)	N/A
Spanish language (primary)	64.6% (135/209)	93.5% (100/107)
Foreign born	Not Available	93.5% (101/108)
COVID-19 infection in household		
Anyone in household previously or currently infected with COVID-19	21.4% (45/210)	38.0% (41/108)

In addition to differences in demographics between the cohorts, participants from HEN/LEAD versus TAB overall had lower socioeconomic status as measured by a number of indicators including household density and food insecurity. Specifically, HEN/LEAD cohorts had a higher number sharing a bathroom and 64.5% of the cohorts experienced food insecurity during the COVID-19 lockdowns versus 34.1% of the TAB cohort (Table 1). Similarly, 55.4% of HEN/LEAD cohorts had household member working during the COVID-19 lockdown in California (3–7/2020) compared with 23.2% of those in the TAB cohort. A high percentage of HEN/LEAD participants are foreign born (93.5%) and of Mexican origin (55.9%). Place of birth was not collected for TAB participants and a lower percentage reported Mexican origin (31.0%). A higher percentage of HEN/LEAD participants reported someone in the household had tested positive for SARS-Co-V2 compared with a household member testing positive in TAB (38.0% versus 21.4%) (Table 1).

### Attitudes towards vaccination against COVID-19 and overall vaccination

A high percentage from both cohorts reported up-to-date vaccination on routine childhood vaccine and influenza with 94.8% of TAB and 95.4% of HEN/LEAD having vaccination up-to-date for self and 94.3% and 97.2% having them up to date for their children respectively (Table 2). Eighty percent of TAB versus a higher 84.3% of HEN/LEAD reported they planned or had already received the vaccine against COVID-19 with a lower 66.7% of TAB and 74.1% of HEN/LEAD reporting that they planned to have their children receive vaccination against COVID-19 (if and when available) (Table 2). These numbers correspond to a 20% and 16.7% nonintent for COVID-19 vaccination for adults in HEN/LEAD and TAB respectively and a 33.3% and 25.9% nonintent for children (Table 2). Approximately, 35.2% of TAB participants and 27.8% of HEN/LEAD stated nonintent for vaccination against COVID-19 for themselves or their children. The most significant reasons for not wanting a vaccine against COVID-19 for self and/or children were (1) concern that the vaccine had not been around for long enough and/or (2) concern about side effects associated with the vaccine (Table 2). Concern about side effects were in general greater for children than adults (Table 2). Both groups reported that news information informed decisions the most about vaccination against COVID-19 with friends ranking second for HEN/LEAD while family was the second biggest influence for TAB (Table 2).

**Table 2:** Attitudes toward COVID-19 vaccination Among Latino households in San Francisco Bay Area

Cohort name	Telomeres at birth (TAB) cohort (n = 211)	Hispanic, eating and nutrition (HEN) & Latino, eating and diabetes (LEAD) cohorts (n = 108)
Vaccine history (including influenza and childhood vaccinations)^^^		
Comfortable with vaccines for self^^^	90.0% (189/210)	87.0% (94/108)
Comfortable with vaccines for children^^^	83.3% (175/210)	90.7% (98/108)
Vaccines up-to-date for self^^^	94.8% (199/210)	95.4% (103/108)
Children vaccines up-to-date^^^	94.3% (198/210)	97.2% (105/108)
COVID-19 vaccine plan		
Wants COVID-19 vaccine for self or has already received vaccine	80.0% (168/210) (*20% refusal)*	84.3% (91/108) (*16.7% refusal*)
Wants child to get COVID-19 vaccine or has already received it	66.7% (70/210) (*33.3% refusal*)	74.1% (80/108) (*25.9% refusal*)
Does not want vaccine for self and/or child	35.2% (74/210)	27.8% (30/108)
Reason for COVID-19 vaccine nonintent		
Vaccine not around long enough	61.9% (26/74)	26.7% (8/30)
Can “survive without vaccine”	21.4% (9/74)	16.7% (5/30)
Does not trust science	14.3% (6/74)	6.7% (2/30)
Does not trust medicine	14.3% (6/74)	3.3% (1/30)
Concern about side effects	35.7% (15/74)	23.3% (7/30)
COVID-19 vaccine side effects		
Fear side effects of COVID-19 vaccine for self (1–10 scale)*	4.4 ± 3.2	5.1 ± 3.4
Fear side effects of COVID-19 vaccine for children (1–10 scale)*	5.5 ± 3.2	6.0 ± 3.45
COVID-19 vaccine influence (self-reported influences/influencers)		
Family	31.0% (65/210)	24.1% (26/108)
Friends	26.7% (56/210)	36.1% (39/108)
Co-workers	21.0% (44/210)	20.4% (22/108)
Social media	15.2% (32/210)	29.6% (32/108)
News media	45.2% (95/210)	41.7% (45/108)

*Higher scores are associated with more fears about side effects

### Risk factors for nonintent to vaccinate against COVID-19

Comparing intent with nonintent for vaccination against COVID-19, we found that mothers who were planning on getting vaccinated were slightly older in TAB (36.2 ± 5.1 versus 34.1 ± 6.3 years; p = 0.01) and for both cohorts, households with greater household person density were at greater risk for nonintent for vaccination against COVID-19 as measured by number of persons sharing bathrooms (4.7 ± 1.8 versus 3.9 ± 1.5; p = 0.048 in HEN/LEAD and 3.8 ± 1.8 versus 3.3 ± 1.6; p = 0.13 in TAB) (Table 3). In TAB, there were also a greater number of total individuals in the house 5.1 ± 2.2 versus 4.5 ± 1.7 (p = 0.09) in families with nonintent versus intent to vaccinate against COVID-19.

**Table 3:** Risk Factors for Not Wanting COVID-19 Vaccine for Self or Child

Variable	TAB (n = 210 Families)		P value	HEN/LEAD (n = 108 Families)		P value
	Plans vaccine or has already received it (n=136)	Vaccine nonintent (n=74)		Plans vaccine or has already received it (n=78)	Vaccine nonintent (n=30)	
Socio-demographics						
Number of children in household	2.0 ± 1.9	2.2 ± 1.1	0.56	2.51 ± 1.01	2.9 ± 1.1	0.12
Mom’s age, years	36.2 ± 5.1	34.1 ± 6.3	**0.01**	35.9 ± 7.1	34.8 ± 6.3	0.49
Married/ living with partner	92.6% (125/135)	89.2% (65/74)	0.40	53.9% (44/78)	44.8% (13/29)	0.41
Socioeconomic and lifestyle variables						
No of people living in the house	4.5 ± 1.7	5.1 ± 2.2	**0.09**	5.1 ± 1.9	5.2 ± 1.5	0.39
No of people sharing bathroom	3.3 ± 1.6	3.8 ± 1.8	0.10	3.9 ± 1.5	4.7 ± 1.8	**0.048**
No of people sharing a bedroom	2.4 ± 1.1	2.5 ± 0.9	0.27	2.39 ± 1.09	2.47 ± 1.01	0.44
Adult working in household during COVID lockdown (yes/no)	77.7% (94/121)	75% (48/64)	0.68	59.1% (39/66)	61.5% (16/26)	0.83
Maternal education level						
High school or less (yes/no)				84.4% (65/77)	89.0% (24/30)	0.58
High school diploma (yes/no)	84.4% (114/135)	77.0% (57/74)	0.18			
Language place of birth and residence						
Spanish predominant	28.2% (38/135)	39.2% (29/74)	0.11	94.9% (74/78)	89.7% (26/29)	0.33
Born in USA	Not available	Not available		3.9% (3/78)	13.3% (4/30)	**0.07**
Years in USA	Not available	Not available		8.1 ± 6.4	9.3 ± 7.5	0.54
Previous COVID infection/experience						
Someone in household previously infected with COVID	16.2% (114/136)	31.1% (23/74)	** < 0.01**	41.0% (32/78)	30.0% (9/30)	0.29
Concern about COVID-19 vaccine side effects^						
For self	3.4 ± 2.7	6.2 ± 3.3	** < 0.01**	4.5 ± 3.2	6.6 ± 3.6	** < 0.01**
For children	4.5 ± 3.0	7.5 ± 2.7	** < 0.01**	5.3 ± 3.2	7.7 ± 3.3	** < 0.01**
COVID vaccine influences						
Family	39.0% (53/136)	16.2% (12/74)	** < 0.01**	30.8% (24/78)	6.7% (2/30)	** < 0.01**
Friends	31.6% (43/136)	17.6% (13/74)	**0.03**	37.2% (29/78)	33.3% (10/30)	0.71
Co-workers	25.8% (35/136)	12.2% (9/74)	**0.02**	23.1% (18/78)	13.3% (4/30)	0.26
News	50% (68/136)	36.5% (27/74)	**0.06**	37.2% (29/78)	53.3% (16/30)	0.13
Social media	15.4% (21/136)	14.9% (11/74)	0.91	28.2% (22/78)	33.3% (10/30)	0.61
Ethnicity						0.94
Central American	44.2% (38/86)	49.0% (25/51)	0.87	38.7% (29/75)	40.7% (11/27)	
Mexican	43.0% (37/86)	33.9% (19/51)		56.0% (42/75)	55.6% (15/27)	
South American/Caribbean/Spain	12.8% (11/86)	13.7% (7/51)		5.3% (4/75)	3.7% (1/27)	
Food insecurity						
Food insecurity score*	1.3 ± 2.6	1.5 ± 2.7	0.34	2.8 ± 3.0	3.1 ± 3.6	0.65
*Food insecurity category*						
High food security	68.6% (83/121)	60.9% (39/64)	0.65	35.8% (24/64)	34.6% (9/26)	0.91
Marginal food security	12.4% (15/121)	15.2% (10/64)		19.4% (13/67)	19.2% (5/26)	
Low food security	13.2% (16/121)	18.8% (12/64)		28.4% (19/67)	38.6% (9/26)	
Very low food security	5.8% (7/121)	4.7% (3/64)		11.5% (11/67)	16.4% (3/26)	

*Higher scores are indicative of greater food insecurity as indicated by the US Household Food Security Survey Module **(**USDA, 2020)

^^^Higher scores indicate greater concern on a scale from 1 to 10

Families who had someone in the household previously test positive for COVID-19 were more likely to have COVID-19 vaccination nonintent in TAB (31.1% versus 16.2%; p < 0.01) although there was no association in HEN/LEAD with prior exposure to COVID-19. For both cohorts, greater concerns about side effects were associated with vaccine nonintent for adults and children (Table 3). Spanish language use in TAB neared statistical significance (p = 0.11) as nonintent was higher in the Spanish-speaking participants versus English-speaking ones (39.2% versus 28.2%). When the data were analyzed just for adult intent to vaccinate themselves in relation to language use, the results were highly significant (34.3% versus 13.3%; p < 0.01) (results not shown). For HEN/LEAD, those that stated they were born in the USA were more likely to have COVID-19 vaccination nonintent, although the results neared statistical significance (p = 0.07; Table 3). Those that planned to get vaccinated against COVID-19 in the TAB cohort were more likely to attribute significant influences from family, friends, co-workers and news sources compared to those with nonintent for vaccination against COVID-19. Influences from family were statistically significant in HEN/LEAD to predict intent to vaccinate against COVID-19 (Table 3).

We found no association between Latino ethnicity, country of origin or food insecurity and risk for nonintent for vaccination against COVID-19 in HEN/LEAD and TAB cohorts (Table 3).

### Qualitative component

For those that answered they would not vaccinate themselves or their children against COVID-19 (n = 74 or 35.2% for TAB and n = 30 27.8% for HEN/LEAD), we asked a question about what would make them more comfortable to vaccinate against COVID-19. Themes that emerged from responses to the qualitative questions for the participants with nonintent suggested that the following factors may change intent regarding vaccination against COVID-19:  (1) more information and research on the vaccines used to prevent COVID-19, (2) more time in the public eye or (3) more assurance from their doctor/health provider that there would be no side effects or that it was safe/needed for their personal health. As all our participants were women of childbearing age, there was a significant amount of concern surrounding potential risk from the COVID-19 vaccine while pregnant, breastfeeding or during the postpartum period and women wanted more assurance and more information that the vaccine used to prevent COVID-19 was safe for women of reproductive age.

## Discussion

We found a high rate of nonintent to vaccinate against COVID-19 in our primarily Latino family cohorts in the San Francisco Bay Area (35.2% in the TAB cohort and 27.8% in HEN/LEAD) comparable with other studies that have focused on Latino communities in the United States [21]. The nonintent to vaccinate against COVID-19 was higher for children in both cohorts (33.3% for children versus 20% adults in the TAB cohort versus 25.9% versus 16.7% in HEN/LEAD as indicated by parental response. This contrasts with the high percentage from both cohorts (> 95%) who are up to date for vaccination for child and self for all other routine vaccinations. These cohorts represent a diverse group of Latino families in the San Francisco Bay Area including a high percentage of foreign-born parents in the HEN/LEAD cohorts as well as socioeconomic heterogeneity in the TAB cohort. Families in HEN/LEAD reported lower socioeconomic status as indicated by maternal education level, employment status and food insecurity compared with those in TAB. At the time of recruitment, HEN/LEAD families participated uniformly in the Special Supplemental Program for Women, Infants and Children (WIC) [14, 15] compared with a heterogenous economic background in TAB. Given the reduced intent to vaccinate against COVID-19 among Latinos in California compared with other population groups and the greater burden of disease in Latinos [2, 20], it is necessary to further disentangle reasons for nonintent to vaccinate against COVID-19 in this population group.

### Age and socioeconomics

Similar to other studies that have found a high rate of vaccine nonintent among younger Latino individuals, we found an overall high rate of vaccine nonintent in all three cohorts (27.8% in HEN/LEAD and 35.2% in TAB). Furthermore, those mothers that were younger in age were more likely to have vaccine nonintent compared with older mothers, specifically in TAB but also in HEN/LEAD although the differences were not statistically significant for HEN/LEAD. It is possible that there is a generational effect and younger Latinas similar to younger other population groups do not feel as vulnerable or at high risk compared with older populations. Alternatively, as other studies have demonstrated, there is less trust of public health institutions and government by younger Latinos than older ones [9].

We also found increased risk for vaccine nonintent among those individuals with greater indicators of crowding but no associations with employment status or food insecurity or other markers of lower socioeconomic status. Previous surveys have found that higher socioeconomic status individuals are more likely to vaccinate using metrics focused on education, income and insurance status [22] or income alone [23]. In our low-income cohorts of primarily foreign-born Latina women (HEN/LEAD), we did not find any association with educational status or employment but rather household density was more predictive of nonintent to vaccinate against COVID-19. Similarly, among the more economically heterogeneous TAB cohort, we also did not find any associations with education or income but crowding indices were also associated with intent to vaccinate against COVID-19. Paradoxically, increased housing density elevates risk for infection with SARS-Co-V2 [17], and our findings suggest that these same high-risk individuals may be less likely to get access to vaccination against COVID-19. Crowding indices may reflect immigration status, which was not collected in our survey, as previous studies have found that undocumented immigrants live in more crowded and less secure housing situations [24]. In TAB, the more socioeconomically heterogenous of the two cohorts, Spanish language use was higher among those not intending to vaccinate, particularly for adult vaccination compared with English language speakers. There was no difference in language use for HEN/LEAD as the population was relatively homogenous linguistically with 93.5% reporting primary Spanish language use.

### Side effects and previous SARS-Co-V2 infection

Greater concern with side effects from vaccination against COVID-19 were significantly associated with nonintent to vaccinate against COVID-19 for all cohorts that were surveyed as similar studies have shown [7]. Future outreach and community-based initiatives need to focus on the minimal risks from vaccination side effects compared with the much larger risk associated with non-vaccination and must be partnered with community approaches and outreach versus marketing campaigns [25]. Interestingly, those participants that had more direct exposure with SARS-Co-V2 infection in the household were less likely to have intent to vaccinate against COVID-19 although this finding was only apparent in the TAB cohort and not HEN/LEAD. It is possible that Latino families have misconceptions about prior exposure to SARS-Co-V2 and need for vaccination (e.g. no need to vaccinate after being exposed). Alternatively, other studies have had participants who voiced desire for physiological immunity as opposed to immunity through vaccination [26]. A multi-ethnic study conducted in the Emergency Department later in 2021, found that parents were more willing to vaccinate children if they had trust in the medical profession [27].

### Influences from family, news and media

In all three cohorts, those individuals that have nonintent to vaccinate against COVID-19 cite fewer influences from family, friends, co-workers and news sources. A large nationally representative Kaiser Family Foundation study found that family and friends were instrumental in persuading individuals to get vaccination against COVID-19 after initial nonintent [28]. Another study with adult women found that exposure to social media may play a positive role in encouraging vaccine uptake [29] and news media exposure may differentially impact Republican versus Democrat-aligned individuals [30]. Similarly, a European study from Greece found that social connections were influential to convince adults to get vaccinated against COVID-19 [31]. To our knowledge, this is the first study focused exclusively on the Latino population in California and suggests the importance of social networks and connections to change nonintent to intent to vaccinate against COVID-19. U.S. Latinos, particularly, those in California, rely on media and family and friends for health-related information [32]. The importance of lay health workers, or trusted members of the community (*promotores/as* in Spanish) to disseminate health information has been well described in the Latino community [33].

### Future directions

Our study has demonstrates the importance of social networks for vaccination intent against COVID-19 including the importance of family, friends and community. We also found increased risk among those in larger families and in higher density living conditions. Key community stakeholders and clinics need to be involved at the grassroots level to dispel myths concerning vaccination and as our study has shown, concern, in particular, surrounding the side effects of vaccination. The US Surgeon general has made a similar recommendation that local “trusted messengers” are key to vaccination against COVID-19 [34]. As described below, since the collection of data for this study, there have been increased efforts to publicize the notion that immigrant status will not play into access to vaccination or enforcement. Community clinics need to better circulate this message as well to ensure that local stakeholders (e.g. *promotores/as*) can disseminate this information widely. As both cohorts had a high percentage of individuals who were up-to-date on vaccination for child and self for all other routine vaccinations including for influenza, this population group is not anti-vaccine, and increased efforts and education and publication will likely result in more vaccination against COVID.

### Limitations

The participants in the present study are part of three, pre-existing, longitudinal cohorts in the Greater San Francisco Bay Area. While our study’s demographics provide insight into reasons for vaccination nonintent among the Latino community, our sample may not be representative of the individual beliefs, values, and behaviors of all United States Latinos. Furthermore, developments in 2021-2 may have altered some perceptions concerning vaccinations. Over the time period that our study was conducted, the United States government and the United States Department of Homeland Security made clear that vaccines will be available free of charge to all individuals in the United States and immigrant status will not play into vaccination nor will enforcement be conducted near clinic sites or sites of vaccination distribution [35]. Furthermore, the Biden administration has made a greater effort to facilitate partnership with Community Health Centers to promote equal access to vaccines among minority population groups [36]. These political changes may have altered attitudes and intent concerning vaccination against COVID-19 after our survey was conducted. Last, during the time period that we administered this survey, only adults were authorized to receive the vaccine against COVID-19 in the United States with 16–17 year old adolescents approved during the last month of our survey for emergency use authorization. As such, our questions about vaccination of children were asked in a hypothetical fashion as vaccination was not yet available for the majority of children in the United States.

## Supplementary Information

**Additional file 1**. Interview questionnaire with questions related to vaccination against COVID-19.

## Data Availability

Data is available via individual request to the PI of the study (Wojcicki).
