# Supplementary material for: Household and social characteristics associated with COVID-19 vaccine intent among Latino families in the San Francisco Bay Area
Source: BMC Infect Dis. 2022 Jun 7;22:527. doi: 10.1186/s12879-022-07467-3 (PMC9171483; doi:10.1186/s12879-022-07467-3)
Supplement: Supplementary file 1 — Additional file 1. Interview questionnaire with questions related to vaccination against COVID-19. [file 12879_2022_7467_MOESM1_ESM.pdf]

COVID Vaccine Questionnaire

Record ID	______________________
Date	______________________
Participant Name	______________________
SNO/Participant ID number	______________________
How Many Children Do You Have?	______________________
What are the ages of your children?	☐ 1<br>☐ 2<br>☐ 3<br>☐ 4<br>☐ 5<br>☐ 6<br>☐ 7<br>☐ 8<br>☐ 9<br>☐ 10<br>☐ 11<br>☐ 12<br>☐ 13<br>☐ 14<br>☐ 15<br>☐ 16<br>☐ 17<br>☐ 18

Do you feel comfortable receiving vaccinations in general?	◯ Yes<br>◯ No
Do you feel comfortable having your children receive vaccinations (all types)?	◯ Yes<br>◯ No
Are you up-to-date with your vaccines?	◯ Yes<br>◯ No
1. Is/Are your child(ren) up-to-date with their vaccines?	◯ Yes<br>◯ No
Have you had COVID-19 infection (confirmed by laboratory testing)?	◯ Yes<br>◯ No
Have any of your children had laboratory confirmed COVID-19 infection?	◯ Yes<br>◯ No
Have any of the other adults in your household had laboratory confirmed COVID-19?	◯ Yes<br>◯ No
Have any of the other children in your household had laboratory confirmed COVID-19?	◯ Yes<br>◯ No
Do you plan or receiving the COVID-19 vaccine when it is offered to you (or have you already received it?)?	◯ Yes<br>◯ No

What are the reasons you are saying no?	☐ You do not trust the science.<br>☐ You do not trust medicine.<br>☐ You think the vaccine hasn't been around long enough.<br>☐ You think you can survive COVID without the vaccine.<br>☐ You don't want the side effects from the vaccine.
Do you plan on allowing your child(ren) to get the COVID-19 vaccine when it becomes available?	◯ Yes<br>◯ No
Has anyone in your household already been vaccinated already with the COVID-19 vaccine?	◯ Yes<br>◯ No
Do your family members influence your views on COVID-19 vaccination?	◯ Yes<br>◯ No
Do your friends influence your views on the COVID-19 vaccine?	◯ Yes<br>◯ No
Do your co-workers influence your views on the COVID-19 vaccine?	◯ Yes<br>◯ No
Does social media impact your views on COVID-19 vaccination?	◯ Yes<br>◯ No
Does the news media impact your views on COVID-19 vaccination?	◯ Yes<br>◯ No
How nervous and concerned about side effects are you to receive the COVID-19 vaccine for yourself (when you become available)? (1 concerned, 10 very concerned)	◯ 1<br>◯ 2<br>◯ 3<br>◯ 4<br>◯ 5<br>◯ 6<br>◯ 7<br>◯ 8<br>◯ 9<br>◯ 10
How nervous and concerned about side effects are you to receive the COVID-19 vaccine for your children (when you become available)? (1 unconcerned, 10 very concerned)	◯ 1<br>◯ 2<br>◯ 3<br>◯ 4<br>◯ 5<br>◯ 6<br>◯ 7<br>◯ 8<br>◯ 9<br>◯ 10
Is there anything which can make you more comfortable with receiving COVID-19 vaccination?	______________________________
Is there anything which can make you more comfortable with receiving COVID-19 vaccination for your children?	______________________________
